# Machine learning-based prediction of mild cognitive impairment among individuals with normal cognitive function

**DOI:** 10.3389/fneur.2024.1352423

**Published:** 2024-02-02

**Authors:** Xia Wei Zhu, Si Bo Liu, Chen Hua Ji, Jin Jie Liu, Chao Huang

**Affiliations:** ^1^School of Computer and Communication Engineering, University of Science and Technology Beijing, Beijing, China; ^2^Intensive Care Unit, Dalian Municipal Central Hospital Affiliated Dalian University of Technology, Dalian, China; ^3^Department of General Medicine, Dalian Municipal Central Hospital Affiliated to Dalian University of Technology, Dalian, China

**Keywords:** dementia, mild cognitive impairment, machine learning, random forest, eXtreme Gradient Boosting

## Abstract

**Background:**

Previous studies mainly focused on risk factors in patients with mild cognitive impairment (MCI) or dementia. The aim of the study was to provide basis for preventing MCI in cognitive normal populations.

**Methods:**

The data came from a longitudinal retrospective study involving individuals with brain magnetic resonance imaging scans, clinical visits, and cognitive assessment with interval of more than 3 years. Multiple machine-learning technologies, including random forest, support vector machine, logistic regression, eXtreme Gradient Boosting, and naïve Bayes, were used to establish a prediction model of a future risk of MCI through a combination of clinical and image variables.

**Results:**

Among these machine learning models; eXtreme Gradient Boosting (XGB) was the best classification model. The classification accuracy of clinical variables was 65.90%, of image variables was 79.54%, of a combination of clinical and image variables was 94.32%. The best result of the combination was an accuracy of 94.32%, a precision of 96.21%, and a recall of 93.08%. XGB with a combination of clinical and image variables had a potential prospect for the risk prediction of MCI. From clinical perspective, the degree of white matter hyperintensity (WMH), especially in the frontal lobe, and the control of systolic blood pressure (SBP) were the most important risk factor for the development of MCI.

**Conclusion:**

The best MCI classification results came from the XGB model with a combination of both clinical and imaging variables. The degree of WMH in the frontal lobe and SBP control were the most important variables in predicting MCI.

## 1 Introduction

Dementia is a syndrome characterized by significantly decreased cognitive function, daily living ability, and social function, which could be caused by various diseases with no reversible or curative treatment. In 2015, an estimated 47 million people age 65 and older were living with dementia, and the number might triple by 2050 (1). Mild cognitive impairment (MCI) is the early stage of dementia and is not linked to any specific etiology (2), which leads to difficulty in early screening of further risk of MCI from populations with normal cognitive function by specific biomarkers. So, it is important to establish a prediction or screening model for the high risk of MCI in populations with normal cognitive function and to provide evidence for exploration of the main MCI etiologies.

During the last 10 years, multiple studies have reported the major risk factors that could promote or predict the development of dementia, including age, education, gender, mental disorder, diabetes, and so on (3, 4). Few focused on the risk or cause of normal cognitive function to MCI. Besides, with the development of imaging, magnetic resonance imaging (MRI) can provide visualizations of different brain lesions such as ischemic strokes, white matter hyperintensity (WMH), and brain atrophy, has increasingly been used for diagnosis and etiology differentiation of cognitive dysfunction (5, 6). Few studies have focused on the combination of both clinical and image factors to determine the future risk of MCI. Compared with a manually completed data analysis by scientists, an artificial model may be more powerful and accurate in detecting the importance of different variables and balancing the weight between each variable and period.

Recently, machine learning algorithms such as neural networks and support vector machines (SVM) have gained significant attention in the field of classification and detection. In 2018, Forouzannezhad et al. (7) proposed SVM with radial basis function in order to detect Alzheimer’s disease, utilizing data from positron emission tomography (PET), MRI, and scores from standard neuropsychological tests. The SVM-based approach achieved a classification accuracy of 81.1% in distinguishing early MCI from normal controls, and an accuracy of 91.9% in differentiating late MCI from normal controls. In 2019, Tingting et al. (8) used effective features derived from functional brain networks to distinguish early MCI from late MCI. They employed five different algorithms for feature selection and SVM for classification and achieved an accuracy of 87.86%.

Convolutional neural networks (CNNs) have easier and more accurate effects on the classification and diagnosis of MCI, and CNNs can automatically find the most discerning disease-related features, which is conducive to avoiding errors introduced by feature engineering. In 2019, the CNNs were used to extract features from MRI for the classification of normal controls, early MCI, and late MCI, and the final accuracy reached 93.96% (binary classification between early MCI and late MCI) and 93.00% (binary classification between normal controls and early MCI), respectively (9). In 2020, Jiang et al. (10) performed a feature selection of structural MRI images through CNNs, and further used SVM to distinguish early MCI from normal controls, achieving an accuracy of 89.4%. In May 2022, El-Sappagh et al. (11) proposed a Long Short Term Memory (LSTM) framework based on information fusion in several patients’ longitudinal multivariate patterns for multi-classification and reached an accuracy of up to 91.22%.

With the above evidence, previous studies mainly focused on machine learning to classify MCI or dementia from normal controls with cross-section image variables, methods on combination of clinical and image variables to find the risk through a dynamic progression from normal cognitive function to MCI have not been well explored. This study aimed to use machine learning technologies including random forest (RF), SVM, logistic regression (LR), eXtreme Gradient Boosting (XGB), and naïve Bayes (NB) to establish a classification model for normal individuals to identify a high future risk of MCI, and to provide evidence for clinicians for the highest clinical and image risk variables of MCI. We used accuracy, recall, and precision as evaluation indicators, to provide a thermotical basis for further deep learning model and etiological analysis.

## 2 Materials and methods

### 2.1 Data collection

The data came from a longitudinal retrospective study involving patients admitted to Dalian Central Municipal Hospital with dynamic cognitive function measurement and MRI examination of at least 3 years apart between 1 January 2008 and 1 January2022. Inclusion criteria: (1) age over 18 years old with a normal baseline cognitive function; (2) patients with dynamic brain image data and detailed clinical data over at least 3 years before a diagnosis of MCI. Exclusion criteria: (1) MCI caused by a special unpreventable factor such as brain tumor, brain trauma, surgery, acute or chronic brain infection, poisoning, paraneoplastic syndrome, drug-related, etc.; (2) dementia caused by acute large vessel infarction or hemorrhage; (3) Subjective cognitive impairment.

Patients were divided into two groups based on the clinical diagnoses at the final visit: the normal cognitive function group (NCF) and the MCI group. The characteristics corresponding to each patient included the following categories (Supplementary Table S1): (1) Clinical variables: including age, education, gender, medical history, and dynamic control of risk factors [including smoking index (number of years smoked * number of cigarettes smoked per day), alcohol consumption index (daily alcohol consumption/100 mL * years), diabetes mellitus years, mean glycated hemoglobin level (Normal, 0: 4–6%; ideal control, 1: 6–7%; under control, 2: 7–8%; poor control, 3: 8–9%; very poor control, 4: ≥9%; 5. unknown), years of hypertension, baseline mean systolic blood pressure (SBP), baseline percentage of SBP over 140 mmHg (%), baseline mean diastolic blood pressure (DBP), baseline percentage of DBP over 90 mmHg (%)]; (2) image variables based on MRI (Philips Achieva 3.0 T magnetic resonance system, Philips Healthcare, United States, 5 mm thick slices): this group of features was classified into continuous features and discrete features. Continuous features were mainly used to quantitative analyze the degree of brain atrophy [defined as: the inner diameter of the forehead angle (mm), the maximum width of the anterior longitudinal fissure (mm), the width of lateral ventricular anterior horn (mm), the index of the lateral ventricular anterior horn (mm), the width of lateral ventricular posterior horn (mm), index of lateral ventricular posterior angle (mm), the width of the third ventricular (mm), index of caudate nucleus (mm), index of lateral ventricular body (mm), mean width of the sulcus (mm), distance of bilateral hippocampal uncinate gyrus (mm), mean width of the hippocampus (mm), mean distance from the temporal lobe to the anterior orbit (mm), minimum width of the middle temporal lobe (mm), mean width of the lateral fissure (mm), maximum transverse width of the midbrain (mm), maximum longitudinal diameter of the midbrain (mm), maximum transverse width of the pons (mm), and maximum longitudinal diameter of the pons (mm)]. Discrete features included the graded brain atrophy of the total brain, degree of total WMH [evaluated through Fazakas score (12)], and WMH regional distribution, which was reported to be independently associated with cognitive dysfunction (6, 13). Atrophy grade was defined as: 0, no atrophy; 1, mild cerebral atrophy, widening and deepening of cerebral sulcus and brain fissure; 2, moderate cerebral atrophy, decreased gyrus volume; 3, severe cerebral atrophy, blade-like gyrus. Paraventricular white matter hyperintensity Fazekas score was defined as 0, no abnormalities; 1, cap-shaped or thin pencil-like; 2, smooth halo; 3, irregular extension to deep white matter. Deep white matter hyperintensity Fazekas score was defined as 0, no abnormality; 1, punctate lesions; 2, the lesion tends to fuse; 3, large-scale fusion of lesions. Total Fazekas score was defined as 0–6 points, scoring the white matter in the paraventricular and deep parts separately, and then adding the scores of the two parts to calculate the total score.

### 2.2 Datasets

The clinical and image variables were classified into 3 datasets. Data set 1 was defined as the clinical variables, including demographic characters, medical history, and previous control of risk factors. Data set 2 was defined as image variables, including the degree and distribution of brain atrophy and WMH. Data set 3 was defined as a combination of clinical and image variables.

### 2.3 Data processing and feature selection

During the data collection process, there were some outliers and missing values. Initially, expert knowledge was used to screen and fill in missing values, especially for important features such as age, education, and hypertension. Considering that the features of each subject were relatively similar, we used forward and backward filling methods to fill in missing values. However, for some records with too many missing values, we chose to delete them directly. Grid search and 5-fold cross-validation were used to determine the optimal number of missing values to be deleted for each model and dataset. If the number of missing values exceeded this threshold, the corresponding records were excluded. For the logistic regression and SVM models, we tried both Min-Max scaling and standardization as normalization methods. We used 5-fold cross-validation to determine the best normalization method for each model.

Employing that too many features not only increased the computational cost but also affected the model’s ability to accurately identify the features that were truly relevant in the predictive model. The wrapper method used an objective function for feature selection, and we adopted the classic Recursive Feature Elimination (RFE) approach. RFE is an iterative process that repeatedly creates models, and at each iteration, it retains the best features or eliminates the worst features. In the next iteration, it used the remaining features from the previous modeling step that were not selected to build the next model. This process continued until all features were exhausted. Then, it ranked the features based on their order of retention or elimination and selected the optimal subset. In our case, we used Random Forest (RF) as the estimator algorithm for the current RFE process. We applied 5-fold cross-validation on the testing dataset and used accuracy as the scoring metric to evaluate the models.

### 2.4 Parameter optimization and evaluation metrics

Grid search is an exhaustive search method that seeks to find the optimal hyperparameters by traversing all possible combinations of hyperparameter values. It began by defining a set of candidate values for each hyperparameter and then generated the Cartesian product of these candidate values, creating a grid of hyperparameter combinations. Subsequently, the model was trained and evaluated for each hyperparameter combination to identify the combination with the best performance. Grid search was used to determine the optimal parameters for each model, performing 5-fold cross-validation and selecting the hyperparameter combination with the highest average accuracy score as the optimal choice.

We pre-processed data by removing missing values, data interpolation, and data normalization, and then used different machine models including RF, SVM, LR, XGB, and NB to classify and compare their accuracy, recall value, precision, and Area Under ROC Curve (AUC), where TP was the number of true positives, TN was the number of true negatives, FP was the number of false positives, and FN was the number of false negatives (see Equations 1–3):


(1) $$\mathrm{Accuracy}=\frac{TP+TN}{TP+FP+TN+FN}$$

(2) $$\mathrm{Precision}=\frac{TP}{TP+FP}$$

(3) $$\mathrm{Recall}=\frac{TP}{TP+FN}$$

Model comparison requires a comprehensive consideration of the “expected generalization performance” of the model in different tasks, which integrates precision and recall. This can be achieved by comparing Receiver Operating Characteristic (ROC) curves. The model generated a real-valued or probability prediction for test samples, which was then compared with a classification threshold. If the prediction value exceeded the threshold, then it was classified as positive; otherwise, it was classified as negative. By sorting the test samples in ascending order, the classification process was equivalent to dividing the samples into two parts using a certain “cut point.” The first part was considered positive, while the second part was considered negative. Multiple thresholds were set within the probability range, resulting in various True Positive Rates (TPR) and False Positive Rates (FPR) values. Plotting the ROC curve using FPR and TPR as the *x* and *y* coordinates, respectively, provided an ROC curve. TPR and FPR were defined as follows:


(4) $$TPR=\frac{TP}{TP+FN}$$

(5) $$FPR=\frac{FP}{TN+FP}$$

Comparing the area under the ROC curve, known as the Area Under the Curve (AUC), allowed for a comprehensive assessment of the expected generalization performance of different models. A larger AUC indicated better generalization performance for the model.

## 3 Introduction to the model

### 3.1 Random forest

Decision tree is a method for classification and regression prediction based on tree structure. A complete decision tree consists of roots, leaves, and internal nodes. The root node is at the top of the decision tree. The leaf node is located at the bottom of the decision tree, storing the results of classification or regression, that is, the prediction results of the decision tree. Internal nodes are nodes for each branch of the decision tree that represent the characteristics selected when splitting nodes.

Random forest (RF) (14), one of the representative algorithms of Bagging, was proposed in 2001 and consists of several independently trained decision trees. RF as a bagging algorithm, using Bootstrap’s sampling idea, randomly put back from m samples to take n samples as training data, and then select t features from all features to establish a decision tree, this process repeated k times to get k decision trees, these k decision trees are combined to get a random forest.

### 3.2 Support vector machine

SVM is a commonly used classification algorithm, which divides data by a hyperplane w^T^x + b: the support vector machine treats the data as independent points distributed in the sample space, and divides the data set by calculating the distance of these points from the hyperplane to find the hyperplane with the “maximum interval.” For nonlinear data, we need to upscale the data and project the data from the original space x into the new space Φ (x), and this method of ascending is the kernel function. There are four common kernel functions, including linear kernel functions, radial basis kernel functions, multiterm kernel functions, and Sigmoid kernel functions. In this work, we used SVM’s default kernel functions, i.e., polynomial kernel functions, and linear kernel functions.

### 3.3 Logistic regression

The logistic regression algorithm is a classic machine learning algorithm, mainly used for classification problems, often used in binary classification. The algorithm works by modeling the relationship between the independent variables and the probability of a particular outcome using the logistic function. This function maps any real-valued number into a value between 0 and 1, which can be interpreted as a probability. In this study, the model calculated the probability that a given input belongs to a certain class and then made a prediction based on a chosen threshold.

### 3.4 eXtreme Gradient Boosting

XGB algorithm proposed by Chen Tianqi (15), which is optimized by the Gradient Boosting Decision Tree (GBDT) algorithm, which is faster and more efficient than the GBDT algorithm, and tends to have higher prediction accuracy in practical applications. The XGB algorithm has two important improvements, the first is to use the Taylor expansion formula to perform a quadratic Taylor expansion of the objective function, which improves the fitting speed and reduces the complexity of the tree. The second is to use regularization to effectively reduce the occurrence of model overfitting problems, and to use the regularization term constructed with the complexity of the tree as the penalty function of the objective function. XGB is widely recognized and used by academia and industry for its excellent training speed and precision.

### 3.5 Naive Bayes

Naive Bayes is a simple yet effective machine learning algorithm based on Bayes’ theorem with an assumption of independence between features. It’s commonly used for classification problems and is particularly efficient when dealing with large datasets. The algorithm calculates the probability of a data point belonging to a certain class based on the presence of particular features. Despite its “naive” assumption of feature independence, Naive Bayes often performs well in practice and is especially suited for text classification tasks, such as spam detection and sentiment analysis. Naive Bayes models are easy to build and can be trained quickly with relatively small amounts of data. They are robust to irrelevant features and work well in multi-class prediction problems. Due to their simplicity and efficiency, Naive Bayes classifiers are frequently used as a baseline for comparison with more complex models, and they can serve as a good starting point for many classification tasks.

## 4 Results

**Figure 1:**
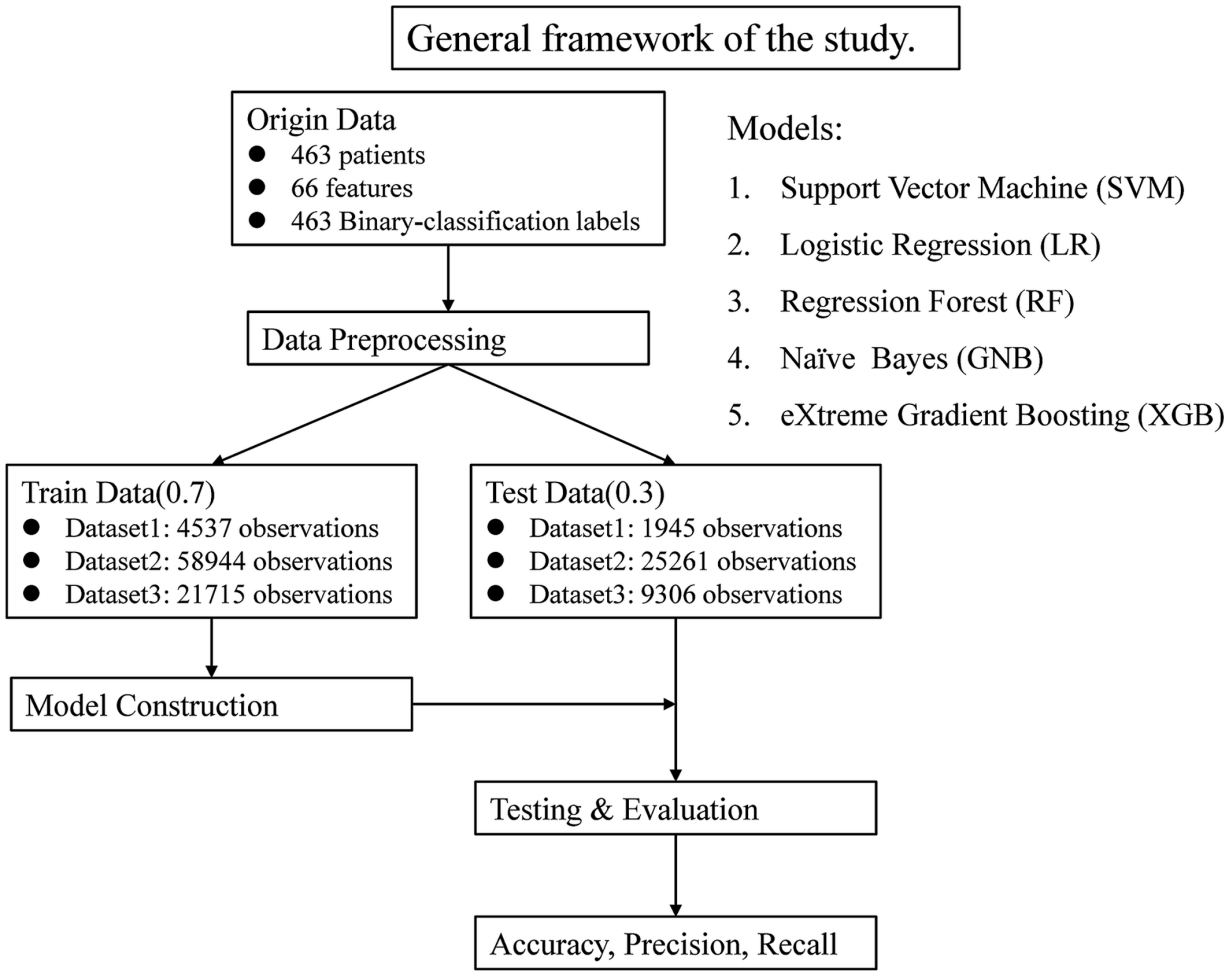
General framework of the study.

There were 463 patients finally involved in this study, including 232 patients with a final diagnosis of NCF and 231 patients with a final diagnosis of MCI. The baseline age was 61.77 ± 8.09 years old in total, and 65.81 ± 8.28 by the last visit, 237 (51.19%) were men, and 264 (57.02%) had higher education. The median interval between baseline to last visit was 6 (4–9) years. All patients had 20 layers of brain MRI images at baseline and the final visit (Table 1). The general framework is shown in Figure 1.

**Figure 2:**
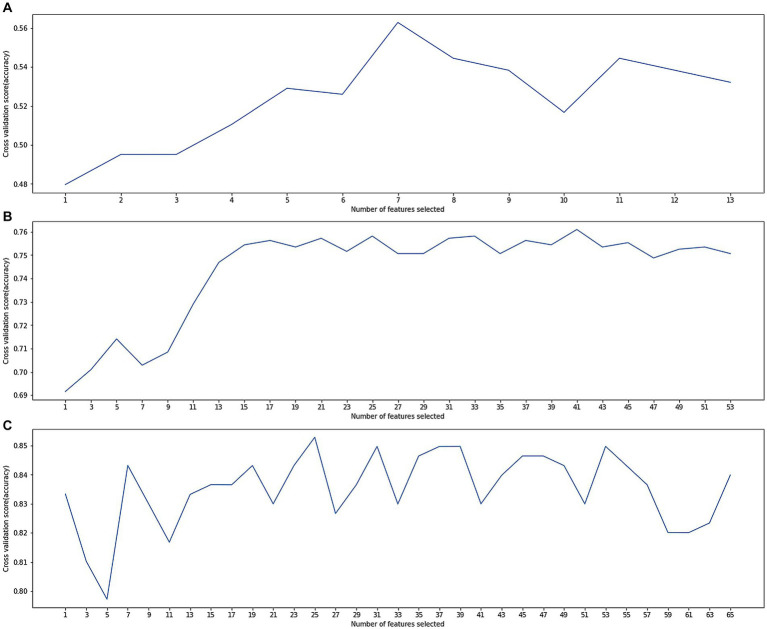
**(A)** Cross-validation score variation along with numbers of selected features in dataset D1; **(B)** cross-validation score variation along with numbers of selected features in dataset D2; **(C)** cross-validation score variation along with numbers of selected features in dataset D3.

The dataset D1 had no outliers or missing values to deal with and feature selection was performed using wrapper methods. As shown in Figure 2A, when selecting the features, the model performance and computing time reached a balance. More features would not increase the model’s accuracy, so these 7 important features were determined: ‘gender’, ‘age at first hospital stay’, ‘degree’, ‘smoking index’, ‘alcohol consumption index’, ‘years of diabetes’, ‘years of hypertension’, ‘mean baseline diastolic blood pressure’, and ‘mean baseline systolic blood pressure’. Subsequently, the model was trained using these 7 features (Table 1).

**Table 1:** Demographic information of individuals with NCF and MCI by last visit.

Groups	Age, years, mean (SD)	Sex, male, *n* (%)		Higher education (*n*, %)	Interval between baseline to last visit, years (IQR)
	Baseline	Last visit	Baseline	Baseline	
Total (*n* = 463)	61.77 ± 8.09	68.31 ± 7.83	237 (51.19%)	264 (57.02%)	6 (4–9)
NCF (*n* = 232)	60.56 ± 7.91	67.31 ± 7.94	117 (50.43%)	162 (69.83%)	7 (4–9)
MCI (*n* = 231)	62.99 ± 8.11	69.32 ± 7.59	120 (51.95%)	102 (44.16%)	6 (4–8)

The results of dataset D1 are shown in Table 2. The accuracy rates of each model including SVM, LSVM, LR, RFC, NB, and XGB were 54.54, 52.57, 65.90, 59.09, 43.18, and 54.54%, respectively. The LR model achieved the best result, with an accuracy of 65.90%.

**Table 2:** The performance of machine learning models under dataset D1.

Models	Precision	Recall	Accuracy
SVM	57.14%	52.17%	54.54%
LSVM	57.89%	45.83%	52.57%
LR	58.33%	73.68%	65.90%
RFC	52.38%	57.89%	59.09%
NB	45.00%	85.71%	43.18%
XGB	52.63%	47.61%	54.54%

SVM, support vector machine; LSVM, linear support vector machine; LR, logistic regression; RF, random forest; NB, Naïve bayes; XGB, eXtreme Gradient Boosting.

The dataset D2 had many anomalies and missing values, which were corrected using expert experience. There were also many missing values, and the optimal number of deleted missing values was determined through a grid search. Records with missing values exceeding this threshold were deleted, and other missing values were filled in by the previous or next record. Feature selection was performed using wrapper methods. As shown in Figure 2B, when selecting 17 features, the model performance and calculation time reached a balance. More features increased computational resource consumption but did not significantly increase the model accuracy. Therefore, these 17 important features were identified:'the widest distance of the forehead angle’, ‘frontal angle index’, ‘mean width of the sulcus’, ‘the maximum distance of the left hippocampal temporal lobe from the anterior orbit’, ‘the shortest distance from the midbrain aqueduct to the ventral anterior edge of the midbrain’, ‘the pontine maximum length diameter’, ‘paraventricular in the frontal lobe’, ‘deep white matter in the frontal lobe’, ‘occipital angle ventricle’, ‘occipital deep white matter’, ‘PVH paraventricular white matter Fazekas score’, ‘DWM Fazekas score’, ‘total Fazekas score’, ‘cerebral small vascular disease total load score’, ‘grade of atrophy of the temporal lobe atrophy’, ‘grade of hippocampal atrophy’, and ‘grade of insular lobe atrophy’. Subsequently, the model was trained using these 17 features.

The results of dataset D2 are shown in Table 3, and the classification accuracy was greatly higher compared with dataset D1, indicating a stronger relationship between image variables and cognitive dysfunction. The accuracy rates of each model including SVM, LSVM, LR, RFC, NB, and XGB were 56.81, 70.55,72.72, 79.54, 56.81, and 70.45%, respectively. The RF also achieved the best classification result, with an accuracy of 79.54%, the precision of 75.0%, and recall of 78.94%.

**Table 3:** The performance of machine learning models under the dataset D2.

Model	Precision	Recall	Accuracy
SVM	52.17%	60.00%	56.81%
LSVM	69.56%	80.00%	70.55%
LR	71.42%	71.42%	72.72%
RF	75.00%	78.94%	79.54%
NB	51.85%	70.00%	56.81%
XGB	67.74%	87.50%	70.45%

SVM, support vector machine; LSVM, linear support vector machine; LR, logistic regression; RF, random forest; NB, Naïve Bayes; XGB, eXtreme Gradient Boosting.

**Figure 3:**
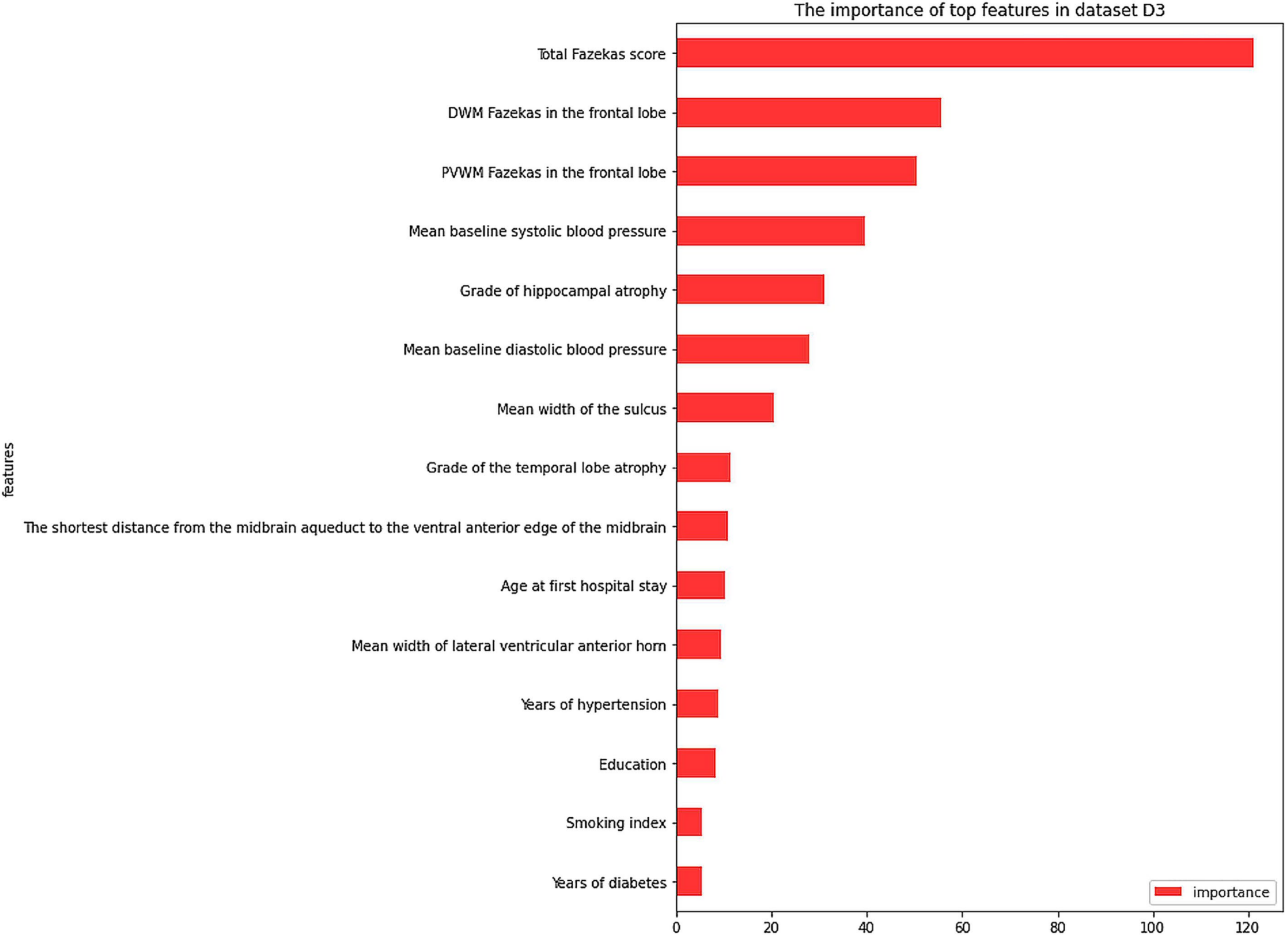
The importance of top features in dataset D3. PVWM, paraventricular white matter; DWM, deep white matter.

The dataset D3 was a combination of D1 and D2, and its anomalies and missing values had been processed in D2. Feature selection was performed using the wrapper methods. As shown in Figure 2C, when selecting 25 features, the model performance and computing time reached a balance. From the clinical aspect, these 15 important features (Figure 3) were identified: ‘total Fazekas score’, ‘DWM Fazekas score in the frontal lobe’, ‘PVWM Fazekas score in the frontal lobe’, ‘mean baseline SBP’, ‘grade of hippocampal atrophy’, mean baseline DBP’, ‘mean width of the sulcus’, ‘grade of the temporal lobe atrophy’, ‘the shortest distance from the midbrain aqueduct to the ventral anterior edge of the midbrain’, ‘age at first hospital stay’, ‘mean width of lateral ventricular anterior horn’, ‘years of hypertension’, ‘education’, ‘smoking index’, and ‘years of diabetes’. Subsequently, the model was trained using these 15 features. The results of dataset D3, which combined the variables of dataset D1 and dataset D2, were shown in Table 4, and the classification accuracy had been greatly higher compared with dataset D1 and dataset D2, indicating a stronger relationship between image variables and cognitive dysfunction. The accuracy rates of each model including SVM, LR, RF, NB, and XGB were 82.96, 89.30, 93.44, 82.31, and 94.32%, respectively. According to Table 4, the best results of dataset D3 had an accuracy of 94.32%, precision of 96.21%, and recall of 93.08%. In addition, the computational complexity of the proposed method was compared in Table 4. On this small-scale dataset, the complexity differences among these models did not affect their performance and usability. From three experimental phases, it had been observed that the training and testing of all models were carried out expeditiously (Where m was the number of features, and n was the number of data).

**Table 4:** The performance of machine learning models under the dataset D3.

Model	Precision	Recall	Accuracy	AUC	Time complexity	Space complexity
NB	95.08%	70.73%	82.31%	0.8324	O(n)	O(m + n)
SVM	88.18%	78.86%	82.96%	0.8329	O(n^2^)	O(n)
LR	91.91%	87.80%	89.30%	0.8942	O(n)	O(n)
XGB	96.21%	93.08%	94.32%	0.9442	O(nlogn)	O(m + n)
RF	96.15%	91.46%	93.44%	0.9361	O(nlogn)	O(m + n)

SVM, support vector machine; LSVM, linear support vector machine; LR, logistic regression; RF, random forest; NB, Naïve Bayes; XGB, eXtreme Gradient Boosting.

**Figure 4:**
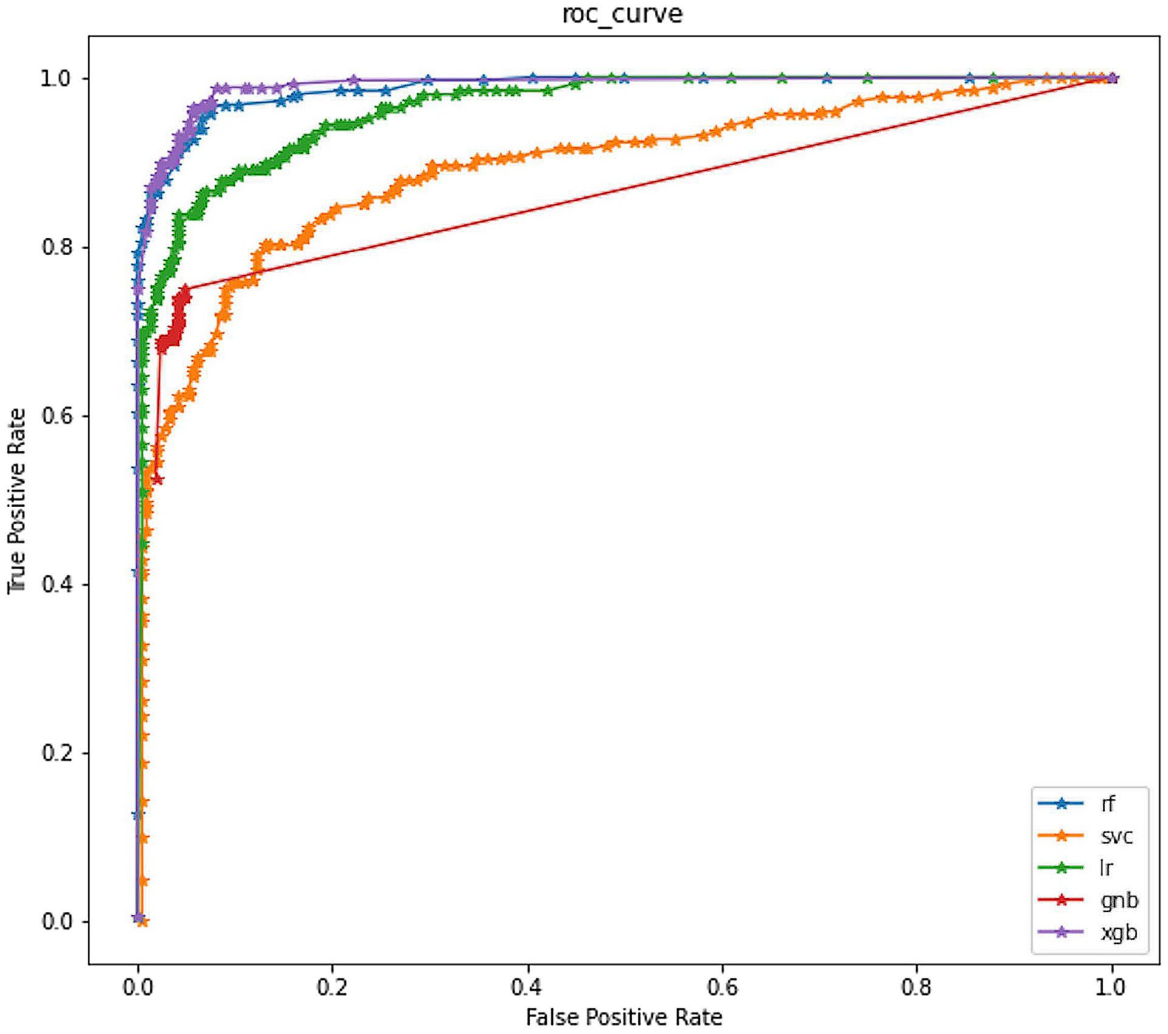
ROC curve of models.

In addition, we also plotted the ROC curve for each model with FPR and TPR as the horizontal and vertical axes, respectively. We calculated the AUC for each model. As shown in Figure 4, the ROC curve of the XGB model almost surrounded that of all other models, and from the perspective of the corresponding AUC, the AUC of the XGB model was also the largest, which was 0.9442.

## 5 Discussion

This study mainly focused on the best MCI prediction models in the cognitive normal population by combining both clinical and imaging parameters and most important etiological factors. Among these machine learning models XGB was the best classification model. The classification accuracy of clinical variables was 65.90%, of image variables was 79.54%, and of a combination of clinical and image variables was 94.32%. The best result of the combination was an accuracy of 94.32%, a precision of 96.21%, and a recall of 93.08%. The XGB model with a combination of clinical and image variables had a potential prospect for the risk prediction of MCI among individuals with normal cognitive function. From a clinical perspective, the degree of white matter hyperintensity, especially in the frontal lobe, and the control of SBP were the most important risk factors for the development of MCI in normal populations.

The best results of the dataset D3 (combination of clinical and imaging parameters) based on the XGB model exceeded the accuracy of most relevant papers that mentioned a diagnosis of MCI in patients with cognitive dysfunction (7, 8, 10, 16). The accuracy of the three reported papers using SVM classification reached 59.2% (16), 81.1% (7), and 87.86% (8), separately. A paper using deep learning based on MCI diagnosis using MRI achieved an accuracy of 89.4% (10).

XGB outperformed other machine learning models such as SVM, LR, RF, and NB in this study due to several advantageous factors. First, XGB employed an ensemble learning approach, combining multiple weak learners into a powerful predictive model that captured complex nonlinear relationships in the data. Second, it utilized gradient boosting, iteratively improving the model by correcting errors made by previous iterations. This iterative process enhanced its predictive capabilities. Third, XGB incorporated regularization techniques, including L1 and L2 regularization, to prevent overfitting and improve generalization on unseen data. Additionally, XGB had built-in mechanisms to handle missing values, automatically learning the best imputation strategy during training. It also provided insights into feature importance, allowing users to identify influential features in the dataset. Lastly, XGB was designed for scalability, supporting parallel processing and distributed computing, making it efficient for large-scale datasets.

Based on dataset D3, the RF model was used to rank the feature importance. The top 5 features of importance were: ‘total Fazekas score’, ‘DWM Fazekas score in the frontal lobe’, ‘PVWM Fazekas score in the frontal lobe’, ‘mean baseline SBP’, and ‘grade of hippocampal atrophy’. These results indicated that a combination of both clinical and image variables was more accurate for the prediction of MCI risk in normal populations. Besides, the top important feature was the degree of total WMH severity and its distribution in the frontal lobe in cognitively normal patients, which was different from the importance of brain atrophy in dementia and the incidence of Alzheimer’s disease (17). However, an increasing number of current studies supported the importance of WMH in patients with dementia (18, 19), even in patients with Alzheimer’s disease (20, 21), similar to the result of our study. This evidence indicated that in patients with normal cognitive function, a higher load of WMH, especially in the frontal lobe, should cause the concern for clinicians regarding a future risk of MCI. Further efforts are needed to understand why the distribution of WMH varies among different lobes of the brain and the potential etiologies, which depend on further automatic segment and measurement of WMH through artificial technology. Secondly, unfavorable control of SBP is another major risk of MCI. Our previous studies have indicated an independent association between blood pressure and WMH (22). This was consistent with the most recently reported randomized control study, the Systolic Blood Pressure Intervention Trial (SPRINT), indicating that intensive SBP reduction to lower than 120 mmHg contributed to a lower progression of WMH load compared with a traditional target of 140 mmHg. So, in cognitively normal patients with hypertension and WMH, intensive reduction of SBP (<120 mmHg) may be the most effective treatment to stop or delay the development of MCI, rather than a traditional control threshold of standard reduction of SBP (<140 mmHg). However, whether higher SBP was associated with the frontal lobe distribution of WMH remains to be explored.

It can be seen that the feature importance ranking of the model was consistent with the clinical studies that explore the association of risk factors and cognitive dysfunction, and further was detailed and comprehensive compared with previous studies (3, 6), which increased the accuracy of the predicting classification of MCI in patients with normal cognitive function. Because doctors’for the most part do not trust machine learning decisions without accurate and powerful explanations (23), our feature importance ranking provided physicians with reliable interpretation of machine learning results (24).

There were several limitations of the study. The first was that we used the Fazekas scale to semi-quantitatively analyze the severity and progression of WMH rather than WMH volume. A more volume quantitative analysis might be more accurate to describe the dynamic change of WMH. The second was that serological or image-specific variables, such as aβ and tau protein, were absent compared to other studies, as the initial purpose of the study was to establish a widely used prediction model in community populations. The third was that a larger sample size and multicenter study may be more representative.

## 6 Conclusion

In this paper, the best MCI classification results came from the XGB model with a combination of both clinical and imaging variables. The distribution of WMH in the frontal lobe and control of SBP was more important than other variables in predicting the risk of MCI in cognitively normal populations. This may bring a new direction to establish a model for fully automatic recognition and segmentation of WMH distribution in the prediction of MCI and etiology analysis. The etiology of WMH distribution variation remained to be explored.

## Data availability statement

The raw data supporting the conclusions of this article will be made available by the authors, without undue reservation.

## Ethics statement

The studies involving humans were approved by the Ethics Committee of the Dalian Central Municipal Hospital (Approval Code: 2023–011-01; Approval Date: 2023.2.16). The studies were conducted in accordance with the local legislation and institutional requirements. Written informed consent for participation was not required from the participants or the participants’ legal guardians/next of kin since the data came from a longitudinal retrospective study.

## Author contributions

XZ: Formal analysis, Investigation, Methodology, Software, Writing – original draft. SL: Formal analysis, Investigation, Methodology, Software, Writing – original draft. CJ: Formal analysis, Investigation, Methodology, Software, Writing – original draft. JL: Conceptualization, Funding acquisition, Project administration, Supervision, Writing – review & editing. CH: Conceptualization, Funding acquisition, Project administration, Supervision, Writing – review & editing.
