# Supplementary material for: Machine learning-based prediction of mild cognitive impairment among individuals with normal cognitive function
Source: Front Neurol. 2024 Feb 2;15:1352423. doi: 10.3389/fneur.2024.1352423 (PMC10870793; doi:10.3389/fneur.2024.1352423)
Supplement: Supplementary file 1 [file Table_1.DOCX]

| **Supplementary table 1. Features and the feature type of the included datasets.** | | | |
| --- | --- | --- | --- |
| Dataset | Features | Feature type | Definition |
| Dataset 1. Clinical variables | Age, years | Continuous features | - |
|  | Education | Discrete features | - |
|  | Gender | Discrete features | - |
|  | History of smoke | Discrete features | - |
|  | Smoking index | Continuous features | Number of years smoked * number of cigarettes smoked per day |
|  | History of alcohol | Discrete features | - |
|  | Alcohol consumption index | Continuous features | Daily alcohol consumption/100ml * years |
|  | History of hypertension | Discrete features | - |
|  | Previous control of hypertension | Continuous features | 1. Years of hypertension; 2. Baseline mean systolic blood pressure (mmHg); 3. Baseline percentage of systolic blood pressure over 140 mmHg (%); 4. Baseline mean diastolic blood pressure (mmHg); 5. Baseline percentage of diastolic blood pressure over 90 mmHg (%) |
|  | History of diabetes | Discrete features | - |
|  | Previous control of diabetes | Continuous features | 1. diabetes mellitus years 2. Previous control of HbA1c: Normal, 0: 4-6%; ideal control, 1: 6-7%; under control, 2: 7-8%; poor control, 3: 8-9%; very poor control,4: ≥9%; 5. Unknown. |
|  | Hyperlipidemia | Discrete features | - |
|  | Hyperhomocysteinemia | Discrete features | - |
|  | Coronary heart disease | Discrete features | - |
|  | Atrial fibrillation | Discrete features | - |
|  | Heart failure | Discrete features | - |
|  | Stroke | Discrete features | - |
|  | Renal dysfunction | Discrete features | - |
|  | Liver dysfunction | Discrete features | - |
|  | Chronic respiratory diseases | Discrete features | - |
|  | Autoimmune diseases | Discrete features | - |
|  | Hyperthyroidism or hypothyroidism | Discrete features | - |
|  | Obstructive sleep apnea syndrome | Discrete features | - |
|  | Anxiety, depression | Discrete features | - |
|  | Sleep disorders | Discrete features | - |
|  | Family history | Discrete features | - |
| Dataset 2. Image variables | Degree of brain atrophy | Discrete features | 1. The inner diameter of the forehead angle (mm), the maximum width of the anterior longitudinal fissure (mm); 2. The width of lateral ventricular anterior horn (mm); 3. The index of the lateral ventricular anterior horn (mm); 4. The width of lateral ventricular posterior horn (mm); 5. Index of lateral ventricular posterior angle (mm); 6. The width of the third ventricular (mm), index of caudate nucleus (mm); 7. Index of lateral ventricular body (mm); 8. Mean width of the sulcus (mm); 9. Distance of bilateral hippocampal uncinate gyrus (mm); 10. Mean width of the hippocampus (mm); 11. Mean distance from the temporal lobe to the anterior orbit (mm); 12. Minimum width of the middle temporal lobe (mm); 13. Mean width of the lateral fissure (mm); 14. Maximum transverse width of the midbrain (mm); 15. Maximum longitudinal diameter of the midbrain (mm); 16. Maximum transverse width of the pons (mm); 17. Maximum longitudinal diameter of the pons (mm). |
|  |  | Discrete features | Graded brain atrophy of the total brain: 0, no atrophy; 1, mild cerebral atrophy, widening and deepening of cerebral sulcus and brain fissure; 2, moderate cerebral atrophy, decreased gyrus volume; 3, severe cerebral atrophy, blade-like gyrus |
|  | Degree of white matter hyperintensity | Discrete features | 1. Paraventricular white matter hyperintensity Fazekas score was defined as: 0, no abnormalities; 1, cap-shaped or thin pencil-like; 2, smooth halo; 3, irregular extension to deep white matter. 2. Deep white matter hyperintensity Fazekas score was defined as: 0, no abnormality; 1, punctate lesions; 2, the lesion tends to fuse; 3, large-scale fusion of lesions. 3. Total Fazekas score was defined as: 0-6 points, scoring the white matter in the paraventricular and deep parts separately, and then adding the scores of the two parts to calculate the total score. |
| Dataset 3. Combination variables | Combination of clinical and image variables | - | - |
